# Poor Diagnostic Performance of the Melanin-Binding Tracer [18 F]MEL050 in Human Melanoma Indicates Biological Heterogeneity

**DOI:** 10.1007/s11307-025-02025-0

**Published:** 2025-06-19

**Authors:** Robert E. Ware, Damien Kee, Peter Roselt, Ivan Greguric, Andrew Katsifis, Thomas Bourdier, Wayne Noonan, William Murray, Catherine Mitchell, Marnie Downes, Mark Shackleton, Grant A. McArthur, Rodney J. Hicks

**Affiliations:** 1 The Melbourne Theranostic Innovation Centre, Level 8, 14-20 Blackwood St, North, Melbourne, VIC 3051 Australia; 2 The Skin and Melanoma Service, the Peter MacCallum Cancer Centre, 305 Grattan St, Melbourne, VIC 3000 Australia; 3 Department of Radiopharmaceutical Sciences, Cancer Imaging, the Peter MacCallum Cancer Centre, 305 Grattan St, Melbourne, VIC 3000 Australia; 4 Australian Nuclear Science and Technology Organisation, Lucas Heights, NSW Australia; 5 Department of Molecular Imaging, Royal Prince Alfred Hospital, Camperdown, Sydney, NSW Australia; 6 Pharmacy School, University of Sydney, Sydney, NSW Australia; 7 The Department of Pathology, the Peter MacCallum Cancer Centre, 305 Grattan St, Melbourne, VIC 3000 Australia; 8 The University of Melbourne, Clinical Epidemiology & Biostatistics Unit, Murdoch Children’s Research Institute, Parkville, Australia; 9 School of Translational Medicine, Monash University, Prahran, VIC 3004 Australia; 10 Department of Oncology, Alfred Health, Prahran, VIC 3004 Australia; 11 Sir Peter MacCallum Department of Oncology, the University of Melbourne, Parkville, Australia; 12 The Department of Medicine, St Vincent’s Medical School, the University of Melbourne, Fitzroy, Australia

**Keywords:** Melanin, PET, Benzamide, Amelanotic melanoma

## Abstract

**Purpose:**

Malignant melanoma is a highly lethal malignancy typically characterized by the expression of melanin, which is an attractive diagnostic and therapeutic target in these cancers because it is expressed in few other tissues. Following preclinical evaluation of the melanin-targeting PET tracer, [18F]-6-fluoro-N-[2-(diethylamino)ethyl] pyridine-3-carboxamide ([18F]MEL050), we sought to evaluate this agent in patients with melanoma.

**Method:**

A phase I clinical trial was performed in ten patients with metastatic melanoma. Safety, dosimetry and diagnostic performance of intravenously administered][18F]MEL050 were evaluated. Based on results from this trial, we further assessed the prevalence and prognostic significance of loss of melanin expression in two historical patient cohorts for which there were matching histological and clinical outcome data.

**Results:**

Across the trial cohort, no adverse safety signals resulted from [18F]MEL050 administration. The whole-body effective dose was 0.0163 mSV/MBq for an adult male and 0.0206 mSV/MBq for an adult female. The human biodistribution was favorable with low uptake in organs at high risk of metastatic spread, including the brain. Of metastatic sites identified as melanoma on [18F]FDG PET/CT, only 31/65 (48%) were positive on [18F]MEL050 PET. Four [18F]FDG+[18F]MEL050+ metastases were resected from three patients and found to be melanotic by histological examination, whereas five [18F]FDG+[18F]MEL050- metastases from two patients were amelanotic. In our historical cohorts, amelanosis was more common in metastatic than primary disease (45% versus 20%) and the presence of melanin within sentinel lymph node metastases was associated with worse disease-free (HR 2.3 95% CI 1.3 - 4.3, *p* = 0.002) and disease-specific survivals (HR 3.6, 95% CI 1.4 - 9.7,*p* = 0.009) in stage III disease, compared with amelanotic sentinel lymph node metastases.

**Conclusion:**

We propose caution in the use of melanin-targeted agents for melanoma diagnosis and therapy until their utility as prognostic or predictive imaging biomarkers, and the biological implications of loss of melanin deposition during melanoma progression, are better understood.

**Supplementary Information:**

The online version contains supplementary material available at 10.1007/s11307-025-02025-0.

## Introduction

Melanomas are derived from melanocytes – highly specialized pigment-producing cells that occur in both cutaneous and non-cutaneous sites, including the eye and mucous membranes. These tumors have a high propensity for dissemination if they are not detected early and completely resected. Recent therapeutic advances, especially the development of V-Raf Murine Sarcoma Viral Oncogene Homolog B (BRAF), mitogen-activated extracellular signal-regulated kinase (MEK) and immune check-point inhibitors, have improved the outlook of patients with stage III and IV melanoma significantly even though many patients still die of their disease [1]. Although there is emerging evidence that neoadjuvant and adjuvant immunotherapy can reduce relapse rates in patients at risk of distant metastasis after resection of a high-risk primary, or of regional nodal metastases, these treatments have significant cost and substantial side-effects that can be both life-threatening and chronic [2]. Accordingly, early diagnosis and treatment of metastatic disease remains important.

While PET scanning with the glucose analogue [^18^F]fluorodeoxyglucose ([18 F]FDG) is now widely used for the staging [3] and surveillance of high-risk melanoma [4], its lack of specificity remains an issue. To address this, several radiolabeled imaging probes that are potentially more specific have been evaluated, primarily using gamma camera imaging. These have included methylene blue dye [5], monoclonal antibodies against melanoma-associated antigens [6], iodoamphetamines [7], α-melanocyte-stimulating hormone analogues [8, 9] and iodinated benzamide (BZA)-based compounds [10–13].

To date, iodinated BZA analogues have been among the most promising melanoma radiotracers because of their high affinity for melanin [14, 15]. Having a very limited distribution in normal tissues, melanin, which is derived by oxidation and polymerization of tyrosine, is an attractive target for the detection of metastatic melanoma since most melanomas continue to produce melanin, thereby presenting a potential disease-specific biomarker. One such radiopharmaceutical with affinity for melanin, N-(2-diethylaminoethyl)−4-[^18^F]fluorobenzamide ([18 F]DAFBA), showed promise for PET imaging [16]. However, its diagnostic potential was found to be compromised in humans by significant hepatobiliary clearance, potentially limiting detection of intra-abdominal disease. Our group, therefore, sought to develop a series of agents with enhanced renal clearance [17]. In pre-clinical evaluation, one of these agents, [^18^F]−6-fluoro-N-[2-(diethylamino)ethyl] pyridine-3-carboxamide ([18 F]MEL050) (Supplementary Figure 1) showed high uptake in melanin containing tissues, very low uptake in amelanotic tumors and highly favorable pharmacokinetic properties [18]. In pigmented B16-F0 xenografts imaged *in vivo*, [18 F]MEL050 achieved target to backgrounds (T:B) of approximately 50:1 at 3 hours post tracer injection, more that 9-fold higher than that of [18 F]FDG.

The highly favorable targeting properties of [18 F]MEL050 encouraged us to perform a first-time-in-human evaluation of this radiotracer. This study aimed primarily to assess the safety, biodistribution, radiation dosimetry and plasma stability of [18 F]MEL050 in participants with recurrent melanoma. Secondary aims were to the assess [18 F]MEL050 uptake in normal melanin containing structures and to determine if non-specific localization occurs in tissues without melanin content. An exploratory aim of the study was to assess the potential of [18 F]MEL050 to determine the extent of detected melanoma metastases in comparison to the contemporary clinical benchmark, [18 F]FDG PET/CT.

We abandoned clinical development of [18 F]MEL050 as this analysis suggested limited clinical utility of melanin imaging for detecting metastatic melanoma. However, these results have become relevant in the context of recent descriptions of closely related probes, including [18 F]−5-FPN, which differs only from [18 F]MEL050 in the position of acylamino and halogen on its pyridine ring [19], and a modified version, [18 F]−5-PFPN, that has also recently been evaluated in humans [20] that suggest that melanin expression may have prognostic implications.

## Study Population and Methods

(See Supplementary Files for a detailed description of methods) In summary, the trial design was a single-center, exploratory micro-dosing study (Phase 0/1) of [18 F]MEL050 using PET/CT imaging in a series of 10 participants known to have metastatic melanoma. The trial was approved by the local human ethics committee (PMCC HREC # 09/045). The Cooperative Research Centre for Biomedical Imaging Development (CRC-BID) was the corporate sponsor of this trial. All patients provided written informed consent.

A histological diagnosis of melanoma, Eastern Cooperative Oncology Group (ECOG) performance score of 0–2, life expectancy of greater than three months, age of 18 years or over, and ability to provide written informed consent were eligibility criteria for the trial. Additionally, entry required at least one site of metastatic disease demonstrated on a [18 F]FDG PET/CT scan performed not more than 2 weeks prior to the [18 F]MEL050 administration, and, to adequately assess for any toxicity related to the investigational agent, lack of systemic anti-melanoma therapy in the 2 weeks before or after [18 F]MEL050 PET/CT scans.

Clinical and laboratory investigations and multiple timepoint PET/CT imaging were performed to further evaluate safety and radiation dosimetry. Whole-body and critical organ radiation exposure was estimated using biodistribution calculations and OLINDA/EXM software.

The study sample size was selected to allow for reasonable detection of any significant adverse events (the study’s primary end-point). The estimated true adverse event rate and its 95% confidence interval were calculated using the modified Wald method. If no adverse events out of 10 are observed, then the 95% CI for the underlying adverse event rate is 0% to 32%. For one adverse event the 95% CI for the underlying adverse event rate is 0% to 43%.

### *Post Hoc* Amelanosis Survey

To assess the clinical, pathological, and prognostic significance of amelanosis, which was a major finding of the clinical phase I trial, two separate cohorts of patients were analyzed as a *post hoc* evaluation. These cohorts of patients had been prospectively enrolled between May 2003 and September 2004 with newly diagnosed primary cutaneous melanoma enrolled at two tertiary melanoma referral centers in Melbourne, Australia. Eligibility criteria for recruitment were:

1. a new diagnosis of primary cutaneous melanoma
2. a formalin fixed paraffin embedded tissue (FFPE) pathological specimen available for review
3. age greater or equal to 16 years, and
4. the ability to provide informed consent.

Approval for this study was obtained from each of the participating center’s ethics committee. These included patients with primary melanoma resection or with positive sentinel lymph node biopsies. Melanin in primary tumors was assessed clinically by patient-reported appearances and visually on microscopy. For the sentinel node series, Schmorl’s staining was performed in addition to routine histopathological evaluation.

## Results

### Imaging Study Population

Twelve patients were enrolled in the study, with 2 failing to meet eligibility criteria. The study population details are detailed in Supplementary Table 1. Participants received a mean of 198 MBq of [18 F]MEL050 (range 165–260 MBq) in 2 ml of normal saline by slow intravenous injection.

### Biodistribution and Radiation Dosimetry

**Fig. 1:**
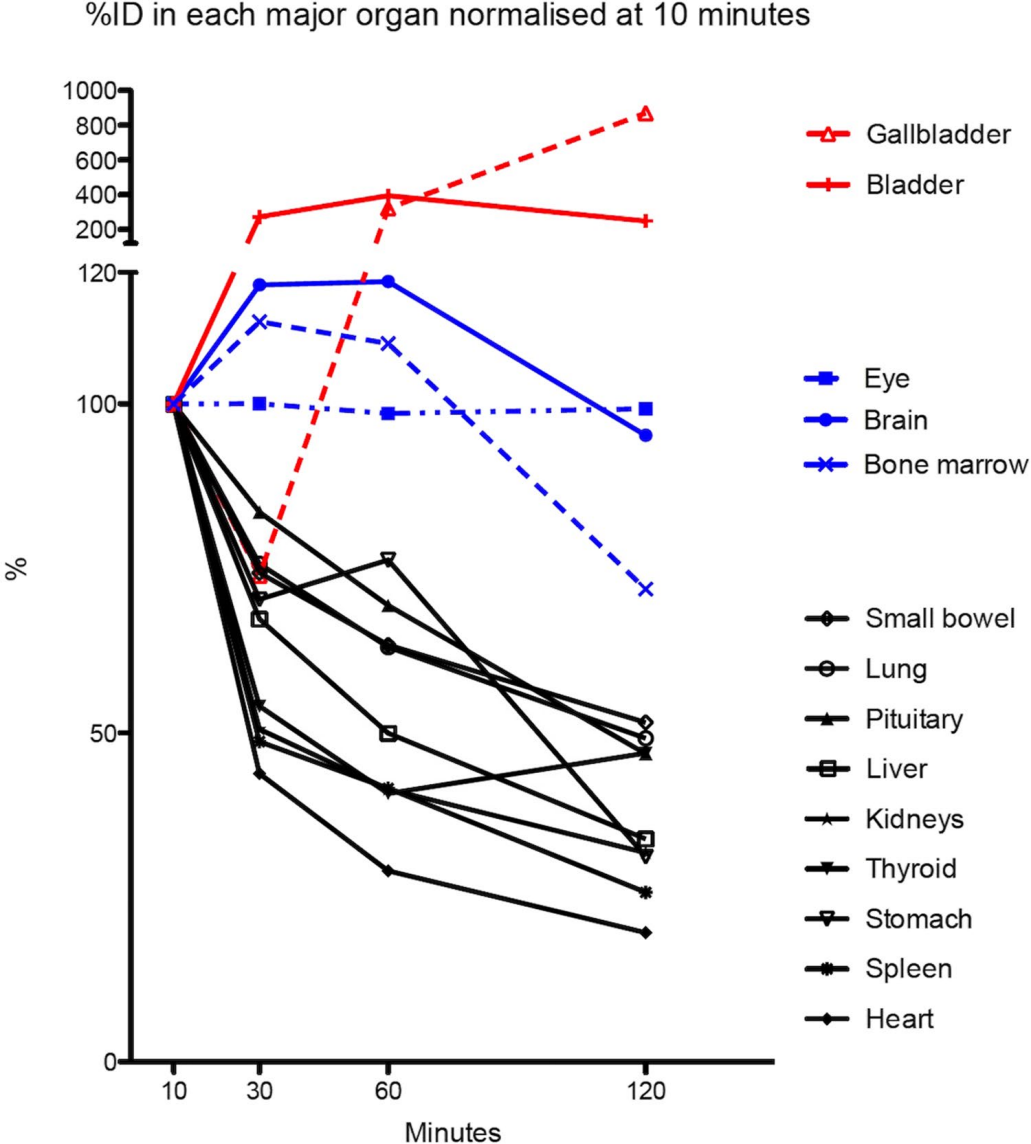
While most tissues demonstrated progressive washout, the eye and bone marrow demonstrated progressive accumulation followed by partial clearance at later timepoints and the bladder, and in some case, gallbladder revealed increasing activity related to tracer clearance

**Fig. 2:**
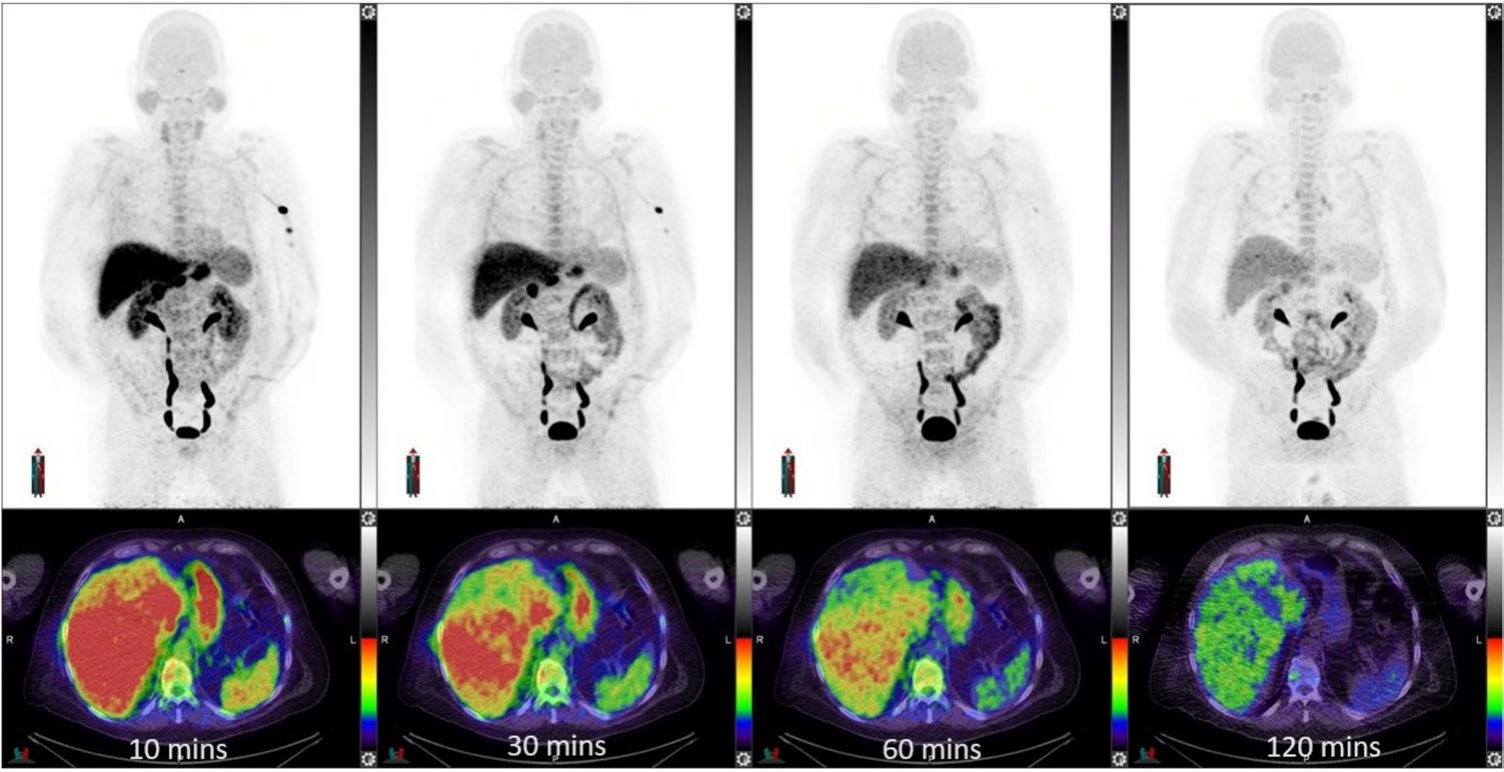
(Patient #8): Sequential maximum intensity projection images (MIP) above and fused PET/CT images below demonstrate early uptake in the liver, spleen and stomach with progressive clearance over time. The MIP images also illustrate early pituitary, salivary gland, and thyroid washout. This patient was very fair skinned with blue eyes and had low uptake in the eyes

Serial [18 F]MEL050 PET imaging analysis showed high initial activity with progressive washout from kidneys, liver, and, in most participants, stomach and pituitary. Low to moderate uptake with little washout occurred in bone marrow and brain. Gastrointestinal tract activity was low in most cases, but gallbladder activity was observed in several patients at late timepoints. Average ocular activity increased from SUVmean 0.95 at 10 minutes to 1.02 at 120 minutes. Qualitative assessment identified 8 participants with definite and 1 with minor specific ocular [18 F]MEL050 localization. (See Supplementary Table 2 for SUVmean values over time and Supplementary Table 3 for percentage of injected dose in reference tissues). The kinetics of uptake and clearance, normalized for activity at 10 minutes after administration of tracer revealed a general trend to clearance of normal tissues but accumulation in some, particularly the eyes and bone marrow (Fig. 1). High early activity in the liver, spleen and stomach demonstrated significant clearance over time (Fig. 2).

Compared to [18 F]FDG, background activity of [18 F]MEL050 was substantially lower in the brain and small bowel, potentially aiding detection of metastases to these sites of known predilection for melanoma spread. Conversely, activity in the liver, stomach and bone marrow were higher at likely scanning times (between 60 and 120 minutes).

The mean dose, normalized for an assumed administered activity of 200 MBq across the cohort of 10 patients was 3.5+/−0.4 mSv (Median 3.3, Range 3.3- 4.1 mSv) (Table 1). The whole-body effective dose was calculated as 0.0163 mSV/MBq for an adult male and 0.0206 mSV/MBq for an adult female, giving a radiation dose of approximately 3.3 mSv and 4.1 mSv for an administered activity of 200 MBq in males and females respectively. Due to the high accumulation of tracer in the urine, the bladder was the critical organ for radiation exposure. This was calculated at 0.88 mSV/MBq assuming a 90-minute bladder void model.

**Table 1:** Radiation dosimetry of MEL050

Absorbed doses of administered ^18^F MEL050	*N*	Mean	SD	Median	Min	Max
Whole body (in milli Sv/200MBq)	10	3.518	0.415	3.260	3.260	4.120
Organ (in micro Sv/MBq)						
Lungs	10	1.957	0.237	1.810	1.810	2.300
Liver	10	1.661	0.227	1.520	1.520	1.990
Spleen	10	0.122	0.012	0.114	0.114	0.139
Thyroid	10	0.692	0.052	0.660	0.660	0.767
Eyes	10	0.000	0.000	0.000	0.000	0.000
Kidneys	10	0.170	0.008	0.165	0.165	0.182
Testes	7	0.000	0.000	0.000	0.000	0.000
Ovaries	3	2.940	0.000	2.940	2.940	2.940
Large intestine	10	1.475	0.169	1.370	1.370	1.720
Small intestine	10	0.097	0.008	0.092	0.092	0.108
Gall bladder	10	0.000	0.000	0.000	0.000	0.000
Urinary bladder	10	4.484	0.715	4.040	4.040	5.520

### Blind Read Designation of Non-Physiological [18 F]MEL050 Activity

All 31 sites judged as representing melanoma on [18 F]MEL050 were confirmed to be true positive based on the combination of pathology and correlative imaging. However, there were no sites judged as pathological that were [18 F]FDG-negative and, therefore, no incremental diagnostic information was obtained from [18 F]MEL050 PET/CT.

Despite a high positive predictive value, [18 F]MEL050 retention that was considered likely to be melanoma was identified in only 31/65 (48%) sites identified on [18 F]FDG PET/CT and was negative for any disease in 3/10 (30%) participants. (See Supplementary Table 4 for details of individual patients), noting that no brain metastases were identified in this cohort.

**Fig. 3:**
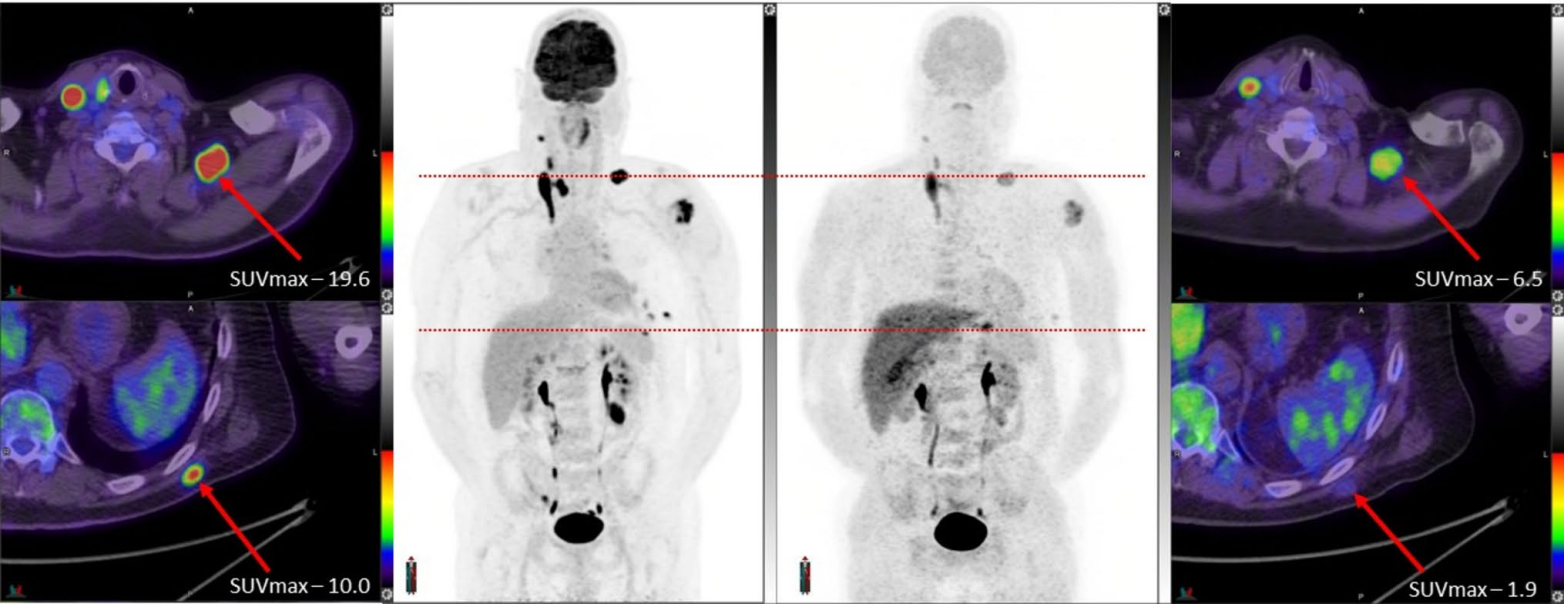
(Patient #9): Fused PET/CT through the lower neck (above) and lower thorax (below) are indicated on the respective MIP images for [18 F]FDG on the left and [18 F]MEL050 on the right. Although [18 F]MEL050 uptake is observed in multiple sites, the uptake in this case is consistently lower than for [18 F]FDG and a subcutaneous lesion in the left lower back was rated as negative on blinded reading

The tumor to background ratio (T/B) averaged 5.4 (range 1.3–11.4) compared to an average of 14.9 (range 1.8–55) for [18 F]FDG. In individual patients, some lesions were true positive, and others were false negative (Fig. 3).

**Fig. 4:**
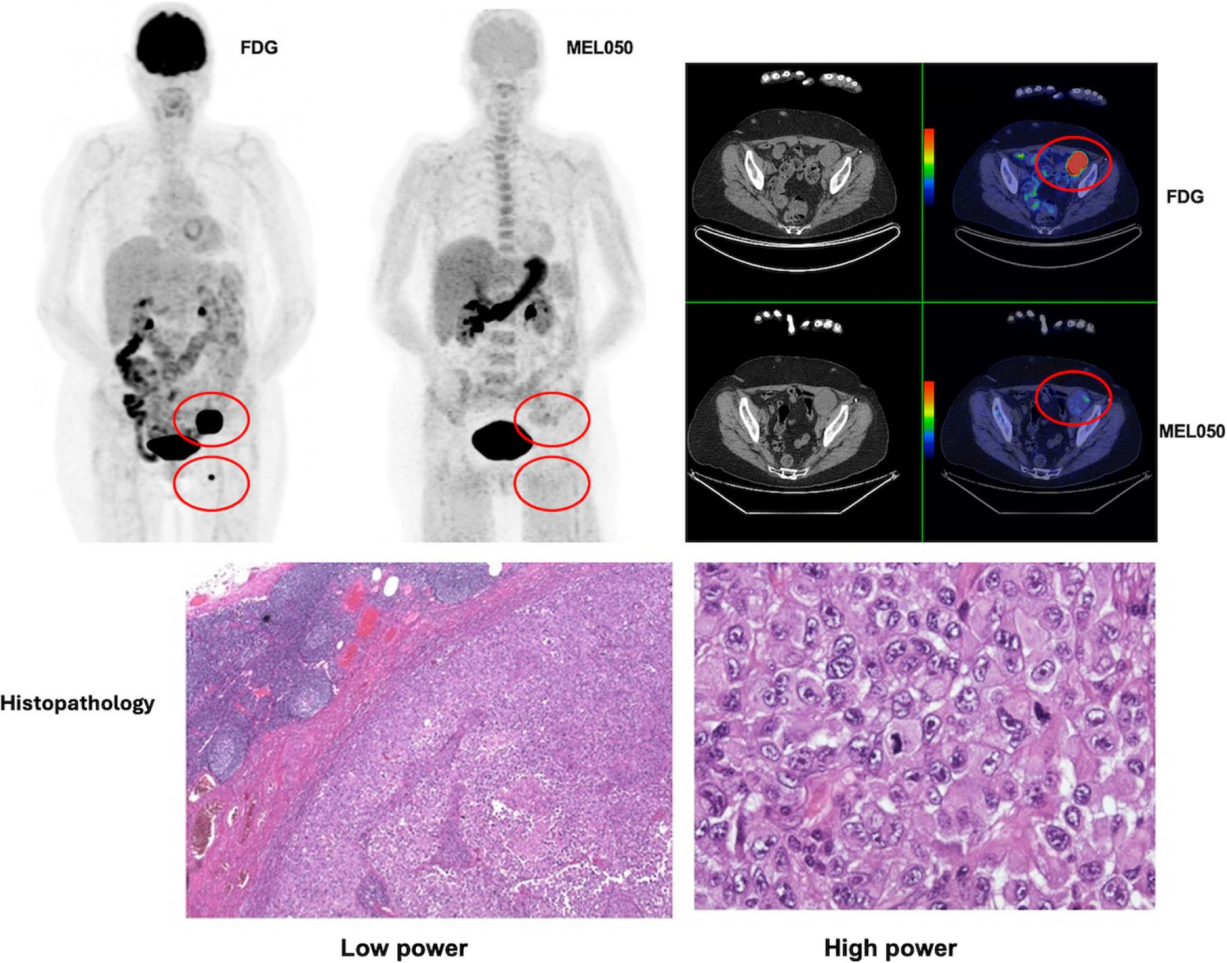
(Patient #1): Left femoral triangle nodal disease subsequently biopsy proven to lack melanin was markedly [18 F]FDG-avid on MIP (far left) and fused transaxial (right upper) but negative on [18 F]MEL050 MIP (centre) and fused PET/CT (right lower). A larger left external iliac node had very patchy [18 F]MEL050 uptake but was primarily negative also

Histological validation of the [18 F]MEL050 PET imaging results was possible for 5 participants. All resected melanotic tumours (4 sites in 3 patients) were true positive on [18 F]MEL050 scans. At 5 sites in 2 patients where [18 F]MEL050 scans were classified as false negative compared to [18 F]FDG, no melanin could be identified in the pathology specimens (Fig. 4). In the remaining 5 patients, rebiopsy to assess melanin staining was not considered appropriate in the context of clear evidence of metastatic disease on both conventional imaging and [18 F]FDG PET/CT.

Follow-up indicated that both [18 F]MEL050 and [18 F]FDG as well as other conventional staging modalities likely underestimated the true extent of disease in that relapse occurred despite curative treatment being attempted through ablation of all known melanoma sites on imaging (5 surgery, 1 radiotherapy). In such cases, further metastases developed 2.5 to 12 months later in 5/6 patients.

### Safety

Patient PMC01 experienced grade 1 increased GGT 20 days after [18 F]MEL050 injection and patient PMC02 experienced grade 1 flushing on the day of the injection. Both adverse events were deemed to be possibly related to the [18 F]MEL050 injection and were resolved without sequalae. No other significant alterations in clinical symptoms, signs, or laboratory values attributable to [18 F]MEL050 administration were recorded.

(Results of follow-up investigations at 28-days and 6-months are included in Supplementary Table 5)

### *Post Hoc* Evaluation of Amelanosis in Clinical Populations

In the first cohort in which the incidence of amelanosis was evaluated in tumors from 253 patients with primary melanoma, 20% were macroscopically amelanotic. As a group, amelanotic melanomas were histologically distinct from pigmented melanomas with the consistent finding of a prognostically more aggressive phenotype. The proportion of amelanotic tumours increased with tumor thickness (*p*_trend_ < 0.001) and, correspondingly, mean Breslow thickness was greater in amelanotic versus pigmented tumours (4.09 mm vs 2.05 mm, *p* < 0.001). Amelanotic primaries were also more likely to be ulcerated (*p* = 0.002), have increased mitoses (*p* < 0.001) and be BRAF wild-type (*p* < 0.001). In a multivariate survival analysis, amelanosis was associated with worse disease-free survival (DFS) (HR 2.3, *p* = 0.031) and disease specific survival (HR 2.5, *p* = 0.033). The 3-year overall survival was 71.2% for amelanotic and 91.4% for pigmented melanomas.

**Fig. 5:**
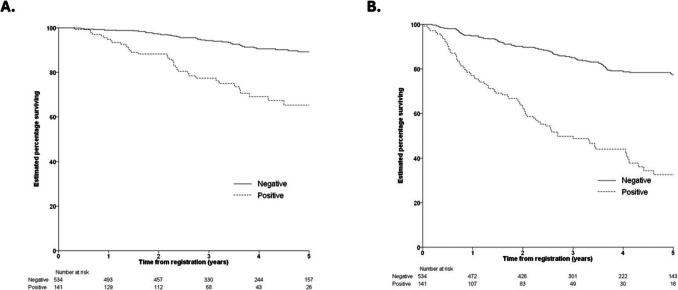
Disease-free survival (**A**) and disease-specific survival (**B**) were significantly worse when sentinel node biopsy samples were positive for melanin on specific staining

The second cohort examined patients with more advanced melanoma. These 142 patients with AJCC 7^th^ edition stage IIIa melanoma had sentinel node tumors examined for melanin. A higher proportion of amelanotic lesions was identified compared with that in primary tumors with 45% being amelanotic. Sentinel node tumor melanin outperformed existing prognostic factors, but contrary to the findings in primary tumors, persistent melanin was associated with worse disease-free (HR 2.3 95% CI 1.3 - 4.3, *p* = 0.002) and disease-specific survival (HR 3.6, 95% CI 1.4 - 9.7, *p* = 0.009) (Fig. 5). Furthermore, in matched biopsies from primary, regional, and distant metastatic sites, the frequency of amelanosis was shown to increase with disease progression – although bidirectional phenotype switching was also observed (Supplementary Figure 2).

## Discussion

At an average administered activity of 198 MBq (range 165–260 MBq), no adverse events of greater than grade 1 severity, or laboratory abnormalities attributable to tracer administration were recorded. This is consistent with the absence of toxicity demonstrated in clinical studies involving structurally related benzamides [19, 21]. The whole-body radiation dose was comparable to those calculated for the current standard PET radiotracer, [18 F]FDG [22, 23]. The bladder had the highest radiation dose, which could be limited by adequate hydration and frequent voiding. Accordingly, there are no safety concerns regarding the use of [18 F]MEL050.

However, in this small series, the diagnostic performance of [18 F]MEL050 was substantially inferior to that with [18 F]FDG, recognizing the potential for a pre-test selection bias with a positive [18 F]FDG PET/CT being required for study entry. Nevertheless, the tumour to background ratios were also generally lower in our series compared to [18 F]FDG.

Despite this, the biodistribution of [18 F]MEL050 was suitable for imaging detection of metastatic sites with predominantly renal excretion, and substantially lower brain and bowel activity than is usual on [18 F]FDG PET scans. Conversely liver, stomach and bone marrow activity remained slightly elevated in comparison to [18 F]FDG at likely clinical scanning times but was still relatively low. Late visualization of the gallbladder suggests the presence of some hepatobiliary excretion of either [18 F]MEL050 or of a metabolite. Even though these differing tissue background activities and excretory patterns could impact on the ability of [18 F]MEL050 PET scans to identify metastases in these locations both positively and negatively, data from this study did not allow a direct evaluation of this possibility with no known brain or gallbladder metastases identified.

The ability of melanin-binding tracers to identify brain metastases that are not apparent on [18 F]FDG PET/CT has recently been demonstrated using a novel benzamide, [18 F]-PFPN, that has structural similarity with [18 F]MEL050 [24]. In a study involving 21 patients imaged with either PET/CT or PET/MRI, this agent demonstrated significantly higher SUVmax values than on [18 F]FDG PET, which translated into improved detection of metastatic sites. The lower intensity of uptake and slightly elevated background activity relative to [18 F]FDG in our series may account for the poor sensitivity of [18 F]MEL050 PET for detection of metastatic sites relative to [18 F]FDG PET/CT.

In contrast, the absence of [18 F]MEL050 accumulation in 5/5 tumours for which pathology demonstrated a lack of pigment, as well as lack of uptake at non-tumoral sites of [18 F]FDG uptake, suggests a high specificity of [18 F]MEL050 for the presence of melanin, as demonstrated in animal models [18]. Furthermore, the detection of definite specific ocular localization of [18 F]MEL050 in 8/10 participants despite substantial partial volume effects related to the minimal thickness of the melanin layer in the retina indicates the capacity of this investigational agent to sensitively detect melanised tissues with PET. Additionally, true positive scans occurred in all 4 tumours in which pathological confirmation of melanin content was available (including 2 tumours with only sparse pigment identified), substantiated the potential of [18 F]MEL050 to identify tissue melanin. Notably, substantial heterogeneity of uptake was noted within individual patients, suggesting loss of melanin in some tumours rather than a low affinity of [18 F]MEL050 for melanin.

Melanomas often have variations in pigmentation. In fact, whereas benign pigmented lesions are generally uniform in colour, colour variegation is a classical sign of melanoma, being the third of four key clinical features first outlined by Friedman in 1985 [25]. Lesions with [18 F]FDG-avidity and lacking uptake of a melanin-targeted tracer have also been recently demonstrated in a larger series using [18 F]PFPN [20]. Clinically, heterogeneity of melanin expression in metastatic lesions may limit the potential of melanin-binding agents for theranostic application, which was one of our motivations for developing a range of iodinated benzamides with structural homology with [18 F]MEL050 [26].

Although amelanosis is thought to occur infrequently in primary melanomas, prompted by the PET findings of this study, we assessed tumour melanin content in a *post hoc* analysis of 253 patients previously prospectively enrolled in an evaluation of primary melanoma specimens and found an amelanosis rate of 18%. In this cohort, amelanosis in primary melanomas was an independent negative predictor of melanoma-specific and relapse-free survival. This may relate to the delayed diagnosis of amelanotic lesions allowing them to become more advanced and therefore more likely to spread. Unfortunately, more detailed staging information, including sentinel lymph node biopsy results, was not available for this cohort to confirm this speculation. However, in the cohort with positive sentinel node biopsy, amelanosis in lymph node metastases was a positive prognostic finding. This finding concurs with a recent study of the melanin-targeted agent, [18 F]PFPN, in which high SUV levels, suggesting a high melanin content, were associated with a poor prognosis [20]. These apparently contradictory findings suggest complex, disease stage-related relationships between melanin deposition by melanoma cells and the metastatic activity of these cells.

Interestingly, in patients with paired pathological samples, amelanosis was more common in nodal metastases than in primary melanomas, suggesting phenotypic plasticity in metastasizing clones, which is a recognized feature of melanoma [27]. Two plastic phenotypic states have been described in melanoma: a proliferative state driven by micropthalmia-associated transcription factor (MITF) and an invasive state linked to upregulation of the tyrosine protein kinase receptor AXL, which is involved in epithelial-mesenchymal transition (EMT). Accordingly, the tendency to metastasize may not necessarily be linked to the rate of progression after metastasis has occurred.

Although the diagnostic performance of [18 F]MEL050 was only an exploratory endpoint of the clinical trial and our evaluation of it was limited by the small sample size of our study, the results discouraged our group from further clinical development of this tracer until such a time as the biological significance of loss of pigmentation in some or all sites of identified disease could be identified. The results of pathological sampling of metastatic nodes suggesting a better prognosis for amelanotic than pigmented lesions support recent findings that a higher SUVmax in tumour sites imaged with [^18^F]PFPN is associated with a superior prognosis [20], but is potentially subject to sampling error and does not necessarily reflect the behaviour of systemic metastases once established.

## Conclusion

While we found that [18 F]MEL050 can be safely administered to humans with biodistribution and radiation dosimetry that is favourable for PET imaging, and that the specificity of [18 F]MEL050 binding for melanoma metastases was high, we also conclude that [18 F]MEL050 has suboptimal sensitivity for detecting melanoma metastases. This is partly due to the heterogeneous expression of melanin in melanoma metastases. Our pathological evaluation of specimens suggests that loss of pigmentation in metastases as opposed to primary melanomas may predict for better outcomes, but these findings warrant further investigation. We propose caution in the use of melanin-targeted agents for melanoma diagnosis and therapy until their utility as prognostic or predictive imaging biomarkers, and the biological implications of loss of melanin deposition during melanoma progression, are better understood.

## Supplementary Information

ESM 1 Supplementary Figure 1 The structure of [18 F]MEL050, which is [18 F]2 from reference [17]. (JPG 82 kb)

ESM 2 Supplementary Figure 2 Longitudinal expression of melanin in serial biopsies derived from individual patients with cutaneous melanoma. Serial expression of melanin in melanoma tumor samples from the retrospect cohort study acquired from the primary tumor, sentinel node, regional node, and distant metastases when available. Tumor specimens derived from a single patient are connected by dashed line and only those with serial samples available are displayed. At each point of analysis, patient samples that remain pigmented or amelanotic throughout disease progression are marked by a circles or squares, respectively. Likewise tumors that either became amelanotic or pigmented upon progression are marked by downward or upward triangles, respectively. (JPG 247 kb)

ESM 3 (DOCX 48 kb)

ESM 4 (DOCX 25 kb)
