# Supplementary figures and images for: Poor Diagnostic Performance of the Melanin-Binding Tracer [18 F]MEL050 in Human Melanoma Indicates Biological Heterogeneity
Source: Mol Imaging Biol. 2025 Jun 19;27(4):649–57. doi: 10.1007/s11307-025-02025-0 (PMC12405299; doi:10.1007/s11307-025-02025-0)

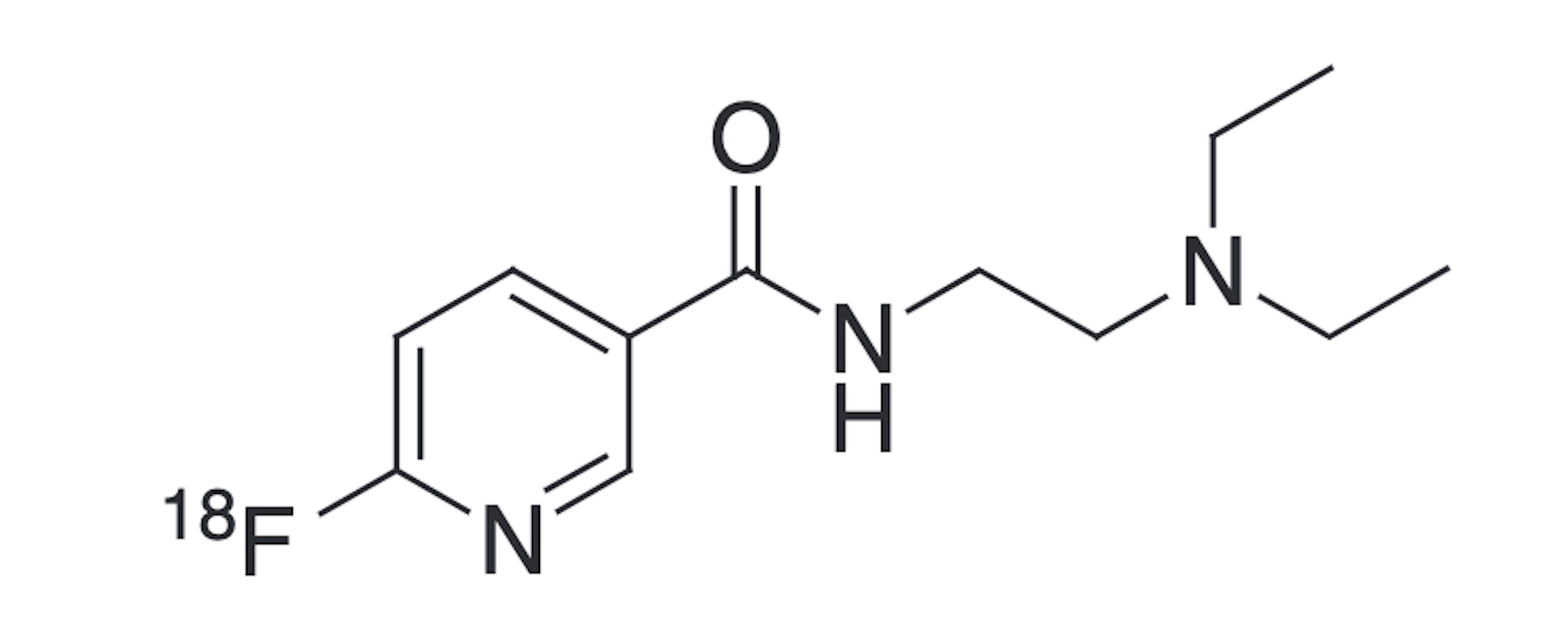

Supplement: Supplementary file 1 — Supplementary Figure 1 The structure of [18 F]MEL050, which is [18 F]2 from reference [17]. (JPG 82 kb) [file 11307_2025_2025_MOESM1_ESM.jpg]

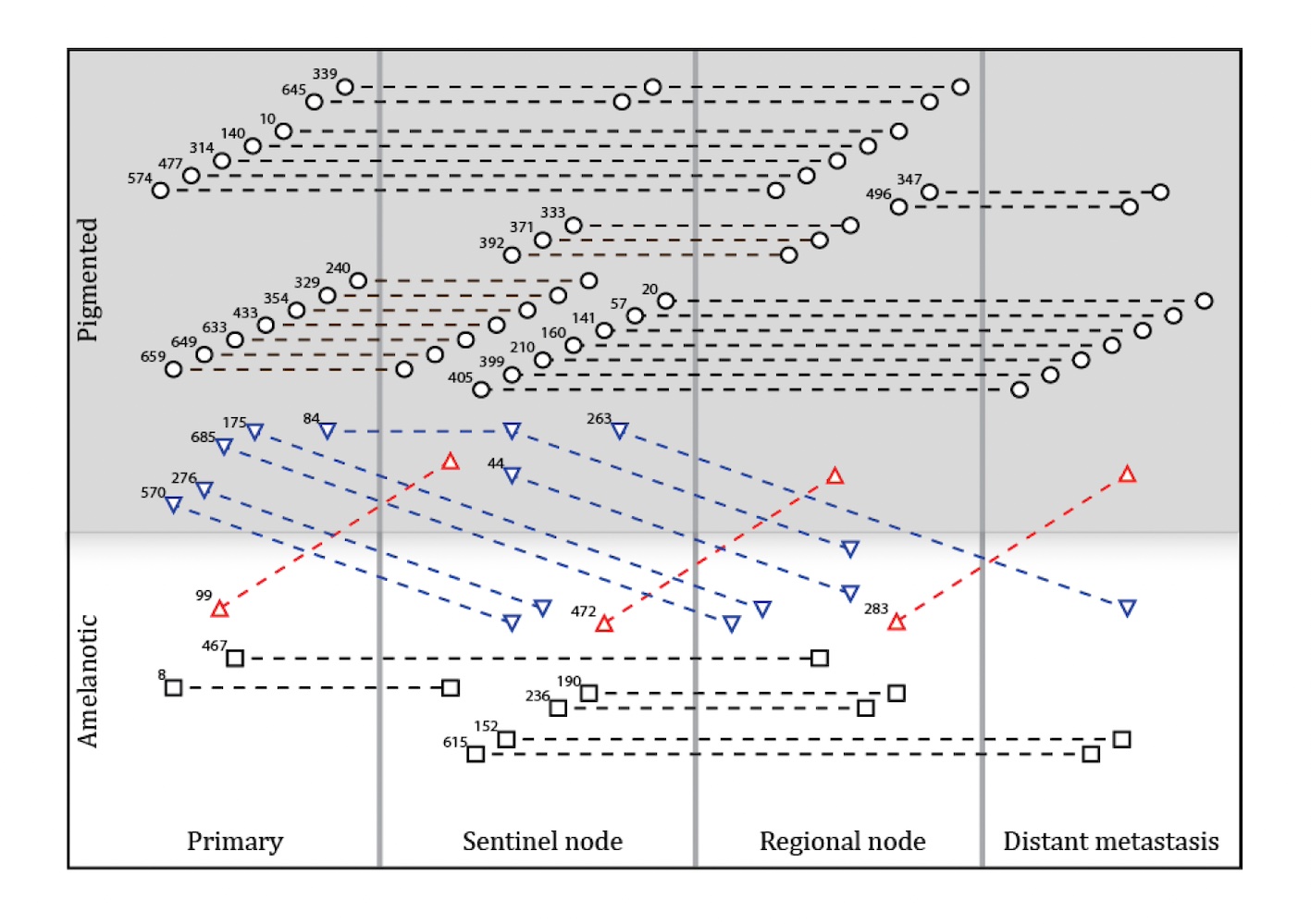

Supplement: Supplementary file 2 — Supplementary Figure 2 Longitudinal expression of melanin in serial biopsies derived from individual patients with cutaneous melanoma. Serial expression of melanin in melanoma tumor samples from the retrospect cohort study acquired from the primary tumor, sentinel node, regional node, and distant metastases when available. Tumor specimens derived from a single patient are connected by dashed line and only those with serial samples available are displayed. At each point of analysis, patient samples that remain pigmented or amelanotic throughout disease progression are marked by a circles or squares, respectively. Likewise tumors that either became amelanotic or pigmented upon progression are marked by downward or upward triangles, respectively. (JPG 247 kb) [file 11307_2025_2025_MOESM2_ESM.jpg]
