# Supplementary material for: Poor Diagnostic Performance of the Melanin-Binding Tracer [18 F]MEL050 in Human Melanoma Indicates Biological Heterogeneity
Source: Mol Imaging Biol. 2025 Jun 19;27(4):649–57. doi: 10.1007/s11307-025-02025-0 (PMC12405299; doi:10.1007/s11307-025-02025-0)
Supplement: Supplementary file 3 — (DOCX 48 kb) [file 11307_2025_2025_MOESM3_ESM.docx]

**Supplementary Table 1- Patient Population**

|  | **All registered patients (*N* = 12)** | | **Patients injected with [18F]MEL050 (*N* = 10)** | |
| --- | --- | --- | --- | --- |
|  | ***N*** | **%** | ***N*** | **%** |
| **Patient characteristics** |  |  |  |  |
| Gender |  |  |  |  |
| Male | 9 | 75% | 7 | 70% |
| Female | 3 | 25% | 3 | 30% |
| Age (in years) |  | |  | |
| Mean | 59.7 | | 59.6 | |
| SD | 9.4 | | 10.4 | |
| Median | 60.0 | | 60.0 | |
| Range | 41 – 74 | | 41 – 74 | |
| Weight (in kg) |  | |  | |
| Mean | 84.9 | | 87.3 | |
| SD | 14.8 | | 14.2 | |
| Median | 83.5 | | 85.5 | |
| Range | 62 – 110 | | 62 – 110 | |
| Height (in cm) |  | |  | |
| Mean | 174.0 | | 173.2 | |
| SD | 7.3 | | 7.6 | |
| Median | 173.5 | | 172.0 | |
| Range | 164 – 189 | | 164 – 189 | |
| BSA (in m^2^) |  | |  | |
| Mean | 2.02 | | 2.04 | |
| SD | 0.19 | | 0.19 | |
| Median | 2.03 | | 2.05 | |
| Range | 1.70 – 2.31 | | 1.70 – 2.31 | |
|  |  |  |  |  |
| **Melanoma medical history** |  |  |  |  |
| Disease stage |  |  |  |  |
| Stage III: sub-stage N1 | 2 | 17% | 2 | 20% |
| Stage III: sub-stage N2 | 2 | 17% | 1 | 10% |
| Stage III: sub-stage N3 | 1 | 8% | 1 | 10% |
| Stage IV: sub-stage M1c | 7 | 58% | 6 | 60% |
| Planned treatment intent |  |  |  |  |
| Curative | 5 | 42% | 4 | 40% |
| Palliative | 6 | 50% | 5 | 50% |
| NA | 1 | 8% | 1 | 10% |

**Supplementary Table 2- SUVmean values over time for study population**

| **mean SUVmean** | 10 | 30 | 60 | 120 |
| --- | --- | --- | --- | --- |
| Bladder | 31.15 | 42.90 | 41.14 | 45.40 |
| Brain | 1.72 | 1.98 | 1.88 | 1.63 |
| Eyes | 0.95 | 0.95 | 0.95 | 1.02 |
| Heart | 3.84 | 2.80 | 2.31 | 1.68 |
| Kidneys | 4.06 | 1.98 | 1.60 | 1.29 |
| Liver | 8.43 | 6.33 | 5.13 | 3.50 |
| Lungs | 3.79 | 2.88 | 2.48 | 2.18 |
| Pituitary | 2.44 | 2.04 | 1.68 | 1.17 |
| Spleen | 5.37 | 3.59 | 2.91 | 2.13 |
| Stomach | 6.97 | 6.17 | 6.53 | 3.76 |
| Thyroid | 2.83 | 2.22 | 1.88 | 1.40 |
| Small bowel | 3.34 | 3.98 | 3.64 | 2.64 |
| Gallbladder | 0.88 | 0.53 | 2.31 | 3.26 |
| Bone marrow | 3.28 | 3.34 | 3.37 | 2.79 |

**Supplementary Table 3- Percentage of Injected Dose in organs of interest**

| **% injected [18F]MEL050 dose detected** | ***N*** | **Mean** | **SD** | **Median** | **Min** | **Max** |
| --- | --- | --- | --- | --- | --- | --- |
| **Lungs** |  |  |  |  |  |  |
| 10 minutes | 10 | 3.78 | 2.45 | 3.57 | 0.76 | 9.40 |
| 30 minutes | 9 | 2.86 | 1.81 | 2.82 | 0.43 | 6.35 |
| 60 minutes | 10 | 2.26 | 1.72 | 2.41 | 0.24 | 6.00 |
| 120 minutes | 10 | 1.78 | 1.42 | 1.82 | 0.20 | 5.18 |
| **Liver** |  |  |  |  |  |  |
| 10 minutes | 10 | 18.12 | 2.50 | 18.23 | 14.81 | 21.46 |
| 30 minutes | 9 | 12.20 | 2.79 | 12.26 | 8.00 | 16.14 |
| 60 minutes | 10 | 9.05 | 1.99 | 8.89 | 5.61 | 12.51 |
| 120 minutes | 10 | 6.14 | 1.53 | 5.97 | 4.08 | 8.83 |
| **Spleen** |  |  |  |  |  |  |
| 10 minutes | 10 | 1.79 | 0.71 | 1.88 | 0.80 | 3.20 |
| 30 minutes | 9 | 0.87 | 0.46 | 0.81 | 0.26 | 1.46 |
| 60 minutes | 10 | 0.74 | 0.31 | 0.77 | 0.32 | 1.21 |
| 120 minutes | 10 | 0.46 | 0.23 | 0.42 | 0.17 | 0.81 |
| **Thyroid** |  |  |  |  |  |  |
| 10 minutes | 10 | 0.10 | 0.05 | 0.11 | 0.00 | 0.18 |
| 30 minutes | 9 | 0.05 | 0.03 | 0.06 | 0.00 | 0.09 |
| 60 minutes | 10 | 0.04 | 0.03 | 0.03 | 0.00 | 0.11 |
| 120 minutes | 9 | 0.05 | 0.03 | 0.05 | 0.00 | 0.09 |
| **Eyes** |  |  |  |  |  |  |
| 10 minutes | 10 | 0.03 | 0.01 | 0.03 | 0.02 | 0.04 |
| 30 minutes | 9 | 0.03 | 0.01 | 0.03 | 0.02 | 0.04 |
| 60 minutes | 10 | 0.03 | 0.01 | 0.03 | 0.02 | 0.04 |
| 120 minutes | 9 | 0.03 | 0.01 | 0.03 | 0.02 | 0.04 |
| **Kidneys** |  |  |  |  |  |  |
| 10 minutes | 10 | 4.22 | 1.28 | 4.18 | 2.76 | 6.61 |
| 30 minutes | 9 | 2.14 | 0.57 | 2.25 | 1.08 | 3.15 |
| 60 minutes | 10 | 1.75 | 0.42 | 1.57 | 1.31 | 2.44 |
| 120 minutes | 10 | 1.34 | 0.42 | 1.26 | 0.81 | 2.13 |
| **Testes** |  |  |  |  |  |  |
| 10 minutes | 0 |  |  |  |  |  |
| 30 minutes | 0 |  |  |  |  |  |
| 60 minutes | 0 |  |  |  |  |  |
| 120 minutes | 0 |  |  |  |  |  |
| **Ovaries** |  |  |  |  |  |  |
| 10 minutes | 1 | 0.03 |  |  |  |  |
| 30 minutes | 1 | 0.03 |  |  |  |  |
| 60 minutes | 1 | 0.03 |  |  |  |  |
| 120 minutes | 1 | 0.03 |  |  |  |  |
| **Large intestine** |  |  |  |  |  |  |
| 10 minutes | 1 | 0.00 |  |  |  |  |
| 30 minutes | 1 | 0.00 |  |  |  |  |
| 60 minutes | 1 | 0.00 |  |  |  |  |
| 120 minutes | 1 | 0.00 |  |  |  |  |
| **Small intestine** |  |  |  |  |  |  |
| 10 minutes | 10 | 2.39 | 2.00 | 1.79 | 0.00 | 6.74 |
| 30 minutes | 9 | 1.97 | 1.45 | 1.66 | 0.73 | 5.18 |
| 60 minutes | 10 | 1.68 | 1.39 | 1.50 | 0.37 | 5.37 |
| 120 minutes | 10 | 1.37 | 0.83 | 1.20 | 0.61 | 3.23 |
| **Gall bladder** |  |  |  |  |  |  |
| 10 minutes | 10 | 0.02 | 0.04 | 0.00 | 0.00 | 0.10 |
| 30 minutes | 9 | 0.01 | 0.04 | 0.00 | 0.00 | 0.12 |
| 60 minutes | 10 | 0.03 | 0.05 | 0.00 | 0.00 | 0.13 |
| 120 minutes | 10 | 0.13 | 0.13 | 0.13 | 0.00 | 0.33 |
| **Urinary bladder** |  |  |  |  |  |  |
| 10 minutes | 10 | 4.31 | 1.85 | 4.55 | 1.30 | 7.21 |
| 30 minutes | 9 | 11.74 | 4.76 | 10.02 | 6.80 | 20.98 |
| 60 minutes | 10 | 17.03 | 5.87 | 15.16 | 9.96 | 27.36 |
| 120 minutes | 10 | 10.72 | 5.85 | 9.76 | 5.64 | 26.18 |

**Supplementary Table 4- Patient Details and Diagnostic Findings**

| **ID** | **Age/Gender** | **Stage** | **[18F]FDG PET/CT** | **[18F]MEL050 PET/CT** | **Melanin** | **Follow-up** |
| --- | --- | --- | --- | --- | --- | --- |
| 1 | 72 F | IIIC | L ext iliac node  L inguinal node | Negative (0/2) | 0, 0% | Distant relapsed @  6.5 months |
| 2 | 64 M | IIIC | R parotid node | R parotid node | 1+,5% | Distant relapsed @  12 months |
| 3 | 42 F | IIIC | R neck nodes (3) | Negative (0/3) | 0, 0% | Local relapse @ 6.5 months |
| 4 | 57 M | IV | R axilla node Cutaneous R flank | R axilla node  Cutaneous R flank | 1+, <1% | 8mm brain met on MRI @ 2.5 months |
| 5 | 66 M | IIIC | R inguinal node | R inguinal node | 2+, 70% | Local relapse @ 6 months |
| 6 | 62 M | IV | R lung  Subcarinal node  R hilar node | Negative (0/3) | NA | Progression of lung and small bowel metastases |
| 7 | 46 F | IV | Bilateral lung (8)  Peri-splenic  Right pelvis | Bilateral lung (3/8)  Other sites negative (0/2) | NA | New bone lesion @5 months |
| 8 | 74 M | IIIC | R axilla nodes (3) | R axilla nodes (1/3) | NA | Radical RT to axilla, CMR@12 months |
| 9 | 57 M | IV | Neck (7)  L chest wall (3)  Abdo/pelvis (4) | Neck (6/7)  L chest wall (1/3)  L arm (1/1)  Abdo/pelvis (1/4) | NA | Ipilimumab, PD@ 3.5 months |
| 10 | 57 M | IV | Extensive (26) | Extensive (14/26) | NA | BRAFi + MEKi, PR |
|  |  |  | Total lesions 65 | 31/65 (48%) |  |  |

Abbreviations: CMR= complete metabolic response (PET); ext = external; F = female; L =left; M = male; NA = not assessed; PD = progressive disease; PR = partial response; R = right; RT = radiotherapy;,(#), Bracketed numbers indicate number of detected lesions at the specified anatomical site.

**Supplementary Table 5- Follow-up Laboratory Testing**

| **Follow-up laboratory tests** | ***N*** | **Mean** | **SD** | **Median** | **Min** | **Max** |
| --- | --- | --- | --- | --- | --- | --- |
|  |  |  |  |  |  |  |
| **HAEMATOLOGY** |  |  |  |  |  |  |
| **Haemoglobin (g/L)** |  |  |  |  |  |  |
| 7 days | 10 | 139.4 | 10.5 | 139.0 | 126 | 155 |
| 28 days | 10 | 136.5 | 11.9 | 134.0 | 117 | 152 |
| 6 months | 9 | 131.3 | 12.6 | 132.0 | 111 | 146 |
|  |  |  |  |  |  |  |
| **WBC (x10^9^/L)** |  |  |  |  |  |  |
| 7 days | 10 | 6.92 | 2.34 | 7.75 | 2.8 | 9.6 |
| 28 days | 10 | 8.13 | 3.55 | 9.05 | 2.8 | 13.0 |
| 6 months | 9 | 5.48 | 1.96 | 5.40 | 3.0 | 8.4 |
|  |  |  |  |  |  |  |
| **Platelets (x10^9^/L)** |  |  |  |  |  |  |
| 7 days | 10 | 283.8 | 51.3 | 273.0 | 215 | 360 |
| 28 days | 10 | 308.2 | 98.5 | 284.5 | 189 | 552 |
| 6 months | 9 | 264.8 | 49.7 | 275.0 | 170 | 328 |
|  |  |  |  |  |  |  |
| **Neutrophils (x10^9^/L)** |  |  |  |  |  |  |
| 7 days | 10 | 4.96 | 1.92 | 5.61 | 1.85 | 7.38 |
| 28 days | 10 | 5.97 | 2.79 | 6.91 | 1.63 | 8.80 |
| 6 months | 9 | 3.89 | 1.72 | 4.17 | 1.89 | 6.98 |
|  |  |  |  |  |  |  |
| **Lymphocytes (x10^9^/L)** |  |  |  |  |  |  |
| 7 days | 10 | 1.29 | 0.45 | 1.20 | 0.58 | 2.01 |
| 28 days | 10 | 1.38 | 0.66 | 1.37 | 0.48 | 2.72 |
| 6 months | 9 | 1.02 | 0.75 | 0.64 | 0.40 | 2.76 |
|  |  |  |  |  |  |  |
| **Eosinophils (x10^9^/L)** |  |  |  |  |  |  |
| 7 days | 10 | 0.22 | 0.12 | 0.21 | 0.04 | 0.43 |
| 28 days | 10 | 0.26 | 0.33 | 0.17 | 0.05 | 1.19 |
| 6 months | 9 | 0.16 | 0.10 | 0.19 | 0.01 | 0.27 |
|  |  |  |  |  |  |  |
| **Basophils (x10^9^/L)** |  |  |  |  |  |  |
| 7 days | 10 | 0.04 | 0.01 | 0.03 | 0.02 | 0.06 |
| 28 days | 10 | 0.04 | 0.02 | 0.04 | 0.02 | 0.08 |
| 6 months | 9 | 0.03 | 0.02 | 0.02 | 0.01 | 0.06 |
|  |  |  |  |  |  |  |
| **BIOCHEMISTRY** |  |  |  |  |  |  |
| **Sodium (mmol/L)** |  |  |  |  |  |  |
| 7 days | 10 | 139.1 | 2.8 | 140.0 | 133 | 142 |
| 28 days | 10 | 139.1 | 3.0 | 139.5 | 132 | 143 |
| 6 months | 9 | 138.1 | 2.8 | 138.0 | 135 | 143 |
|  |  |  |  |  |  |  |
| **Potassium (mmol/L)** |  |  |  |  |  |  |
| 7 days | 10 | 4.47 | 0.33 | 4.55 | 3.9 | 4.9 |
| 28 days | 10 | 4.38 | 0.46 | 4.40 | 3.7 | 5.1 |
| 6 months | 9 | 4.44 | 0.36 | 4.40 | 4.0 | 4.9 |
|  |  |  |  |  |  |  |
| **Chloride (mmol/L)** |  |  |  |  |  |  |
| 7 days | 6 | 103.8 | 3.6 | 104.0 | 99 | 109 |
| 28 days | 5 | 101.8 | 2.2 | 102.0 | 99 | 105 |
| 6 months | 2 | 103.0 | 2.8 | 103.0 | 101 | 105 |
|  |  |  |  |  |  |  |
| **Urea (mmol/L)** |  |  |  |  |  |  |
| 7 days | 10 | 5.91 | 2.07 | 5.65 | 3.2 | 10.3 |
| 28 days | 10 | 5.46 | 1.40 | 5.35 | 3.7 | 7.2 |
| 6 months | 8 | 6.80 | 2.01 | 6.10 | 4.8 | 11.0 |
|  |  |  |  |  |  |  |
| **Creatinine (umol/L)** |  |  |  |  |  |  |
| 7 days | 10 | 84.3 | 13.3 | 80.5 | 64 | 108 |
| 28 days | 10 | 83.4 | 16.9 | 78.5 | 57 | 112 |
| 6 months | 9 | 81.3 | 17.1 | 82.0 | 57 | 111 |
|  |  |  |  |  |  |  |
| **Random blood glucose (mmol/L)** |  |  |  |  |  |  |
| 7 days | 10 | 5.60 | 1.25 | 5.00 | 4.3 | 8.3 |
| 28 days | 10 | 5.43 | 0.88 | 5.10 | 4.2 | 6.6 |
| 6 months | 5 | 6.26 | 2.25 | 5.60 | 4.6 | 10.2 |
|  |  |  |  |  |  |  |
| **Calcium (mmol/L)** |  |  |  |  |  |  |
| 7 days | 9 | 2.38 | 0.10 | 2.39 | 2.19 | 2.51 |
| 28 days | 10 | 2.39 | 0.06 | 2.41 | 2.28 | 2.49 |
| 6 months | 7 | 2.35 | 0.13 | 2.37 | 2.09 | 2.52 |
|  |  |  |  |  |  |  |
| **Bilirubin (umol/L)** |  |  |  |  |  |  |
| 7 days | 10 | 8.90 | 2.42 | 8.00 | 6.0 | 14.0 |
| 28 days | 10 | 8.30 | 2.58 | 8.00 | 5.0 | 13.0 |
| 6 months | 9 | 8.89 | 4.01 | 8.00 | 5.0 | 18.0 |
|  |  |  |  |  |  |  |
| **ALP (U/L)** |  |  |  |  |  |  |
| 7 days | 10 | 84.6 | 10.4 | 81.0 | 71 | 107 |
| 28 days | 10 | 88.0 | 11.7 | 85.0 | 71 | 112 |
| 6 months | 9 | 87.8 | 16.7 | 81.0 | 72 | 120 |
|  |  |  |  |  |  |  |
| **GGT (U/L)** |  |  |  |  |  |  |
| 7 days | 10 | 35.2 | 23.4 | 26.0 | 15 | 86 |
| 28 days | 10 | 41.3 | 23.5 | 35.5 | 13 | 84 |
| 6 months | 9 | 38.4 | 23.6 | 33.0 | 16 | 91 |
|  |  |  |  |  |  |  |
| **ALT (U/L)** |  |  |  |  |  |  |
| 7 days | 10 | 21.9 | 9.4 | 17.0 | 12 | 38 |
| 28 days | 10 | 24.7 | 7.4 | 27.0 | 12 | 35 |
| 6 months | 9 | 23.0 | 8.9 | 23.0 | 14 | 38 |
|  |  |  |  |  |  |  |
| **AST (U/L)** |  |  |  |  |  |  |
| 7 days | 0 |  |  |  |  |  |
| 28 days | 0 |  |  |  |  |  |
| 6 months | 1 | 24.0 |  |  |  |  |
|  |  |  |  |  |  |  |
| **Protein (g/L)** |  |  |  |  |  |  |
| 7 days | 10 | 71.3 | 6.9 | 69.5 | 63 | 84 |
| 28 days | 10 | 71.0 | 6.0 | 70.0 | 63 | 84 |
| 6 months | 9 | 70.3 | 7.9 | 69.0 | 58 | 85 |
|  |  |  |  |  |  |  |
| **Albumin (g/L)** |  |  |  |  |  |  |
| 7 days | 10 | 43.2 | 2.7 | 42.5 | 40 | 49 |
| 28 days | 10 | 43.0 | 2.1 | 43.0 | 39 | 46 |
| 6 months | 9 | 41.6 | 3.3 | 42.0 | 36 | 46 |
|  |  |  |  |  |  |  |
| **CLOTTING** |  |  |  |  |  |  |
| **INR** |  |  |  |  |  |  |
| 7 days | 9 | 1.20 | 0.64 | 1.00 | 0.9 | 2.9 |
| 28 days | 8 | 1.10 | 0.37 | 1.00 | 0.9 | 2.0 |
| 6 months | 7 | 1.16 | 0.51 | 1.00 | 0.9 | 2.3 |
|  |  |  |  |  |  |  |
| **APTT (s)** |  |  |  |  |  |  |
| 7 days | 9 | 30.3 | 3.7 | 28.0 | 28 | 38 |
| 28 days | 8 | 30.4 | 4.0 | 30.5 | 24 | 36 |
| 6 months | 7 | 29.9 | 3.2 | 29.0 | 26 | 35 |
|  |  |  |  |  |  |  |
