# Supplementary material for: Poor Diagnostic Performance of the Melanin-Binding Tracer [18 F]MEL050 in Human Melanoma Indicates Biological Heterogeneity
Source: Mol Imaging Biol. 2025 Jun 19;27(4):649–57. doi: 10.1007/s11307-025-02025-0 (PMC12405299; doi:10.1007/s11307-025-02025-0)
Supplement: Supplementary file 4 — (DOCX 25 kb) [file 11307_2025_2025_MOESM4_ESM.docx]

**Supplementary Methods**

Safety Assessments

Safety was assessed by a trial management committee including an independent assessor not involved in the trial directly in sequential cohorts of 1, 1, 1, 3 and 4 patients, with a minimum of 7 days between each cohort and graded according to the Common Criteria for Adverse Events (CTCAE) version 4.0. Initiation of each cohort occurred only after the approval of the safety committee following review of the previous cohort’s 7-day safety outcomes.

Radiopharmaceutical Quality Control

The investigational agent was extemporaneously compounded for each trial participant under Good Manufacturing Practice (GMP) conditions by Cyclotek Australia Pty. Ltd. and comprised a sterile, apyrogenic injection with a specific activity averaging 127+/- 65.5 GBq/μmol and radiochemical purity of greater than 95%.

Clinical and Laboratory Evaluation

Prior to administration, a full medical history and examination with baseline hematology, and biochemistry were performed. Following administration of the investigational agent safety was evaluated by monitoring vital signs regularly for 3 hours along with arterial oxygen saturation and repeat biochemistry and hematology laboratory investigations. Clinical and laboratory parameters were also evaluated at 24 and 168 hours after injection of the investigational product. Any adverse events were recorded with causation assessed by clinicians experienced in routine care of patients with melanoma.

In 6 participants, blood samples were obtained at 1, 5, 20, 75, 115 and 180 minutes post-injection along with urine measurements at 90 and 180 minutes post injection for assessment of total radioactivity and tracer breakdown. In 4 participants only urine samples for total radioactivity excretion and 180 minute blood samples for standard laboratory evaluation were obtained.

PET/CT Acquisition

The prescribed dose of 200 MBq was prepared onsite and dispensed by a nuclear medicine technologist using a dose calibrator. It was administered by slow intravenous push through a secure cannula. A low-dose, non-contrast CT scan (140 kVp, 40-200 mAs, GE smart mA with final slice thickness 3.27mm) encompassing the vertex to the lower thighs was acquired followed by serial 3D PET acquisitions on a GE STE-8 PET/CT (GE Medical System, Milwaukee, WI USA) encompassing the vertex to the lower thighs (8 bed positions of 1, 1.5 and 2 minutes at 10, 30 and 60 minutes post-injection, respectively) without patient movement. At 90 minutes post-injection the patient ambulated and voided and at 120 minutes post-injection the participant commenced a further low dose CT and PET scan (3 minutes/ bed position as described above). PET scans were reconstructed with 2 iterations, 20 subsets, 3mm filter, 128 x 128 matrix, 55mm reconstructed transaxial FOV and final slice thickness 3.27 mm.

Biodistribution. Visual assessment was supplemented with semi-quantitative assessment of major organ retention at 4 time points. Manually defined threshold-adapted regions of interest were defined for major organs using combined CT and PET images to define mean organ standard uptake values (SUV), metabolic volume and percentage of the injected dose (%ID) in various organs.

Radiation Dosimetry. Measurements obtained in the biodistribution assessment were input in OLINDA/EXM software [1] to obtain estimates of major organ and whole body dosimetry. The 90-minute bladder voiding model was implemented.

MEL050 retention in the eyes, and pathological tissues was assessed using qualitative and semi-quantitative measurements as described above.

Lesion Identification

The definition of pathological lesions for further analysis involved providing 2 experienced nuclear medicine physicians with the calculated group mean organ biodistribution data, as well as each participant’s MEL050 PET scan series.

Blinded to disease status and scan type, each physician then randomly viewed 20 de-identified PET/CT image sets, comprising the 60 minute FDG and MEL050 studies for each participant. Sites of non-physiological uptake were identified by each observer and designated as exaggerated physiological, pathological but non-melanoma, or melanoma. Sites concordantly designated as melanoma were subject to semi-quantitative analysis with measurement of SUVmax and SUVmean, metabolic volume, % ID and tumor to background ratio (T/B).

Scan accuracy was only an exploratory objective so formal histopathological sampling of all sites deemed as melanoma was not undertaken. FDG PET/CT was used as the standard of truth unless subsequent clinical pathological or imaging data indicated otherwise. MEL050 and FDG PET/CT scan findings were designated as True Positive (TP), True Negative (TN), False Positive (FP) or False Negative (FN) on a per lesion and per patient basis according to the above criteria.

Evaluation of Melanin Content

Patients with resectable melanoma who underwent subsequent resection of FDG PET-defined melanoma metastases had excised tissue histologically assessed using Schmorl’s stain for melanin (performed routinely by the Department of Anatomical Pathology, Peter MacCallum Cancer Centre) and assessed by an experienced pathologist blinded to MEL050 scan results. Melanin expression was scored based on the intensity of melanin stain using a 4-point scale (score of 0-3) and the percentage of cells involved (0-100%).

Amelanosis Survey

A previously established cohort of patients prospectively enrolled between May 2003 and September 2004 with newly diagnosed primary cutaneous melanoma that had been enrolled at two tertiary melanoma referral centers (the Alfred Hospital and the Peter MacCallum Cancer Centre) in Melbourne, Australia was analyzed *post hoc* to assess the incidence of amelanotic primary lesions. Eligibility criteria for recruitment were:

1. a new diagnosis of primary cutaneous melanoma

2. a formalin fixed paraffin embedded tissue (FFPE) pathological specimen available for review

3. age greater or equal to 16 years, and

4. the ability to provide informed consent.

Approval for this study was obtained from each of the participating center’s ethics committee.

Amelanosis in this analysis was macroscopically determined using two independent methods. Firstly, patient-defined amelanosis was extracted from a structured questionnaire regarding their melanoma’s appearance performed at the time of initial patient enrolment. Coloration described as either pink, red, purple, or pearly white was classified as amelanotic. Second, pathology determined amelanosis was defined as the lack of visible melanin pigmentation based on macroscopic examination of the excised tumor sample by laboratory scientist and extracted from the original pathology report.

For the second cohort, consecutive patients who had undergone sentinel node evaluation following a diagnosis of primary melanoma from 1 January 2000 until 31 April 2010 were identified from an electronic database at the Peter MacCallum Cancer Centre. Patient demographics, primary tumor characteristics, sentinel node biopsy and any subsequent completion lymph node dissection details, and clinical outcome data were collected from their medical record. Demographic data included age at sentinel node biopsy and gender. Primary tumor characteristics were recorded as the most advanced state of disease from either the original primary tumor excision report (which may have been performed at an external pathology laboratory) or subsequent wide local excision. Where central review of an externally reported specimen was available, this information was used in preference. We recorded Breslow thickness, ulceration, mitoses, Clark level, histological subtype, tumor-infiltrating lymphocytes, regression, lymphovascular or perineural invasion and satellitosis. For sentinel node and completion lymph node dissection the total number and number of positive nodes were recorded. Follow-up data obtained included (i.) date and site of first melanoma relapse; (ii.) date of death; and (iii.) cause of death (melanoma or other cause). Where patients had simultaneous relapse of metastases (local, regional, or distant) the most advanced disease site was recorded. For patients with less than two years of available follow-up contemporary information was sought from their local medical practitioner.

For all patients with a positive sentinel node result we retrieved archived slides (H&E, S100) and FFPE tumor blocks. Additional 4 micron sections were cut and stained for Ki-67 (Cell Marque SP6 clone, 1:50 dilution) and with Schmorl’s stain for melanin by a laboratory scientist (Department of Anatomical Pathology). All prepared slides were centrally reported using a standardized template by an experienced melanoma pathologist who was blinded to patient outcomes. The histological features assessed were: single maximum dimension of the largest tumor deposit; the tumor penetrative depth (S-classification) [2]; tumor localization (using the Dewar classification) (Dewar et al., 2004 [3]); presence of extracapsular extension; melanin expression; and Ki-67 score. Assessment of melanin content was performed using three methods. From H&E stained slides, melanin was assessed by the percentage of cells containing melanin and also by using the method as described by Viros et al. [4]. From the Schmorl stained slides melanin was assessed by both the percentage of cells containing melanin and intensity of stain (4 point scale). A score of 0 on this scale was considered as amelanotic. The presence or absence of melanophages was recorded separately as determined from viewing H&E stained sections. Ki-67 was scored as a percentage of tumor cells stained.

Analysis was performed using Stata Statistical Software release 10 (Stata Corporation, Texas, USA) and GraphPad Prism version 5.0 (GraphPad Software, California, USA). Non-random relationships between patient and tumor characteristics and the presence of amelanosis were examined using a univariate logistic regression model and Fisher’s exact test. Patient survival outcomes were defined as follows:

1. Disease-free survival (DFS) was calculated from the date of primary

melanoma excision until the date of first melanoma relapse, excluding any local relapse or the development of a new primary melanoma.

2. Overall survival (OS) was calculated from the date of primary melanoma

excision until the date of death from any cause.

3. Disease-specific survival (DSS) was calculated from the date of primary

melanoma excision until the date of death from melanoma.

4. Patients without a defined survival end-point were censored at the date of

last known follow-up; or, for the analysis of DSS, at the time of death from a

non-melanoma cause.

Survival curves for amelanotic and pigmented melanomas were generated for DFS, OS and DSS using the Kaplan-Meier method and compared using the log-rank (Mantel-Cox) test. A Cox proportional hazards regression was performed to examine for an independent effect on survival of amelanosis in comparison to other known prognostic factors. For all statistical tests, a threshold of p < 0.05 was used to define statistical significance.

**References**

1. Stabin, M.G., R.B. Sparks, and E. Crowe, *OLINDA/EXM: the second-generation personal computer software for internal dose assessment in nuclear medicine.* J Nucl Med, 2005. **46**(6): p. 1023-7.

2. Starz, H., K. Siedlecki, and B.R. Balda, *Sentinel lymphonodectomy and s-classification: a successful strategy for better prediction and improvement of outcome of melanoma.* Ann Surg Oncol, 2004. **11**(3 Suppl): p. 162S-8S.

3. Dewar, D.J., et al., *The microanatomic location of metastatic melanoma in sentinel lymph nodes predicts nonsentinel lymph node involvement.* J Clin Oncol, 2004. **22**(16): p. 3345-9.

4. Viros, A., et al., *Improving melanoma classification by integrating genetic and morphologic features.* PLoS Med, 2008. **5**(6): p. e120.
